# Adalimumab safety and mortality rates from global clinical trials of six immune-mediated inflammatory diseases

**DOI:** 10.1136/ard.2008.102103

**Published:** 2009-01-14

**Authors:** G R Burmester, P Mease, B A C Dijkmans, K Gordon, D Lovell, R Panaccione, J Perez, A L Pangan

**Affiliations:** 1 Charité-University Medicine Berlin, Free University and Humboldt University of Berlin, Berlin, Germany; 2 Swedish Medical Center, Seattle, Washington, USA; 3 VU Medical Centre and Jan van Breemen Institute, Amsterdam, The Netherlands; 4 Evanston Northwestern Healthcare, Skokie, Illinois, USA; 5 Cincinnati Children’s Hospital Medical Center, Cincinnati, Ohio, USA; 6 University of Calgary, Calgary, Alberta, Canada; 7 Immunology Development, Abbott Laboratories, Parsippany, New Jersey, USA; 8 Abbott Laboratories, Abbott Park, Illinois, USA

## Abstract

**Objectives:**

Clinical trials of tumour necrosis factor antagonists have raised questions about the potential risk of certain serious adverse events (SAE). To assess the safety of adalimumab in rheumatoid arthritis (RA) over time and across five other immune-mediated inflammatory diseases and to compare adalimumab malignancy and mortality rates with data on the general population.

**Methods:**

This analysis included 19 041 patients exposed to adalimumab in 36 global clinical trials in RA, psoriatic arthritis (PsA), ankylosing spondylitis (AS), Crohn’s disease (CD), psoriasis and juvenile idiopathic arthritis (JIA) to 15 April 2007. Events per 100 patient-years were calculated using SAE reported after the first dose to 70 days after the last dose. Standardised incidence rates were calculated for malignancies using national and state-specific databases. Standardised mortality rates (SMR) were calculated for each disease using data from the World Health Organization.

**Results:**

Cumulative rates of SAE of interest in RA have remained stable over time. Rates of SAE of interest for PsA, AS, CD, psoriasis and JIA were similar to or lower than rates for RA. Overall malignancy rates for adalimumab-treated patients were as expected for the general population. SMR across all six diseases indicated that no more deaths occurred with adalimumab than expected in the general population.

**Conclusions:**

Based on 10 years of clinical trial experience across six diseases, this safety report and the established efficacy of adalimumab in these diseases provide the foundation for a better understanding of its benefit–risk profile.

Tumour necrosis factor (TNF) plays an important role in the pathogenesis of rheumatoid arthritis (RA), juvenile idiopathic arthritis (JIA), psoriatic arthritis (PsA), ankylosing spondylitis (AS), Crohn’s disease (CD) and psoriasis. Anti-TNF therapies have proved effective in these diseases, either as monotherapy or in combination with other immunosuppressive therapies. All three commercially available TNF antagonists, adalimumab (Humira; Abbott Laboratories, Abbott Park, Illinois, USA), etanercept (Enbrel; Immunex, Thousand Oaks, California, USA) and infliximab (Remicade; Centocor, Inc, Malvern, Pennsylvania, USA), are indicated for RA, PsA, AS and psoriasis.^1^ ^2^ ^3^ ^4^ ^5^ ^6^ ^7^ ^8^ ^9^ ^10^ ^11^ ^12^ ^13^ ^14^ ^15^ Adalimumab and infliximab are also indicated for CD; adalimumab and etanercept are indicated for JIA.^16^ ^17^ ^18^ ^19^ ^20^ ^21^

Based on the results of clinical trials,^1^ ^2^ ^3^ ^4^ ^5^ ^6^ ^7^ ^8^ ^9^ ^10^ ^11^ ^12^ ^16^ ^17^ ^18^ ^19^ ^20^ ^21^ questions have arisen about the potential risk of serious infections, including tuberculosis, and malignancies with TNF antagonists.^22^ ^23^ ^24^ Other adverse events (AE) of interest that have been reported following TNF antagonist treatment include lupus-like syndromes, demyelinating disorders and congestive heart failure (CHF).^25^ ^26^ ^27^ ^28^ ^29^ Long-term data from an extensive number of patients with different immune-mediated inflammatory diseases can provide further insight into the safety of TNF blockade.

Adalimumab, the first fully human monoclonal antibody targeted against TNF, was first administered to a study patient in 1997.^30^ We evaluated safety data from approximately 10 years of clinical trial experience with adalimumab in six diseases. Our primary objectives were to: (1) extend the results of the initial RA clinical trial safety analysis by Schiff *et al*^31^ through two additional years, evaluating the stability of serious adverse event (SAE) rates over time; (2) evaluate and compare the safety of adalimumab in PsA, AS, JIA, CD and psoriasis within the context of the established safety profile in RA; and (3) evaluate malignancies and mortality rates from adalimumab clinical trials and compare them with rates from the general population. This report represents the largest clinical trial dataset of the three TNF antagonists, and is the first comprehensive safety analysis of a TNF antagonist across six immune-mediated inflammatory diseases.

## Methods

Data were derived from 36 global clinical trials of adalimumab, 19 in RA, three in PsA, three in AS, one in JIA, five in CD and five in psoriasis, including randomised controlled trials, open-label trials and long-term extension studies to 15 April 2007. Inclusion criteria for these trials ensured that the index disease was present and that patients had active disease appropriate for clinical trial enrolment. Exclusion criteria were generally standardised and included: the presence of clinically active tuberculosis, active listeriosis, acute or chronic hepatitis B, or a history of hepatitis C; persistent or severe infections requiring hospitalisation or treatment with intravenous antibiotics 30 days before baseline or oral antibiotics within 14 days of baseline; a history of neurological symptoms suggestive of a demyelinating disorder; a significant history of cardiac, renal, neurological, psychiatric, endocrinological, metabolic, or hepatic disease that would adversely affect participation in the study; a history of malignancy other than carcinoma in situ of the cervix or successfully treated, non-metastatic squamous or basal cell skin carcinoma and a known history of HIV infection.

### Rates of SAE of interest

Investigators reported AE occurring after the first dose of adalimumab up to 70 days (five half-lives) after the last study dose. SAE were defined as those that were fatal or life threatening, those that required inpatient hospitalisation or prolongation of existing hospitalisation and those that resulted in persistent or significant disability or required medical/surgical intervention to prevent another serious outcome.^32^ If a patient gave birth to a child with a congenital anomaly or birth defect, experienced a miscarriage, or underwent an elective abortion, these were also labelled SAE. SAE were coded using the Medical Dictionary for Regulatory Activities (MedRA).

This analysis focused on generally accepted events of interest for the anti-TNF class, which include serious infections, tuberculosis, opportunistic infections, demyelinating disorders, lupus-like syndrome, CHF, malignancies, lymphomas and non-melanoma skin cancer (NMSC). Predetermined search criteria were used to identify events in these categories, and all cases underwent medical review. For each disease, SAE rates were reported as events per 100 patient-years (number of events divided by the total patient-years of exposure and multiplied by 100).

### Malignancy and mortality data versus the general population

Standardised incidence rates (SIR) were calculated using the ratio of the observed number of cancers to the expected number of cancers for each cancer site. 95% CI for the SIR were calculated based on the Poisson distribution.^33^ The expected numbers of cancers for SIR calculations were based on two data sources: (1) 5-year age-specific cancer incidence rates obtained from the National Cancer Institute (NCI) Surveillance Epidemiology and End Results database (1993–2001) for all cancers other than NMSC^34^ and (2) 10-year age-specific incidence rates for NMSC (basal cell carcinoma (BCC) and squamous cell carcinoma (SCC)) from an NCI survey of eight locations in the USA (1977–8).^35^ Sensitivity analyses were performed using expected NMSC rates from two other sources: BCC and SCC incidence rates in Arizona (1996) and SCC incidence rates in Minnesota (1984–92), states with considerably discordant skin cancer rates.^36^ ^37^

A standardised mortality rate (SMR), the ratio of observed deaths to expected deaths, for each disease was calculated using the expected rates based on country-specific age and sex-matched general population data from the World Health Organization to 2002.^38^ Deaths during clinical trials, whether related to the study drug or not, were included in the analysis.

## Results

Baseline characteristics of patients are provided in table 1. A total of 19 041 patients received adalimumab. Of these, 12 345 were patients with RA, of whom 1472 received adalimumab therapy for at least 5 years. Rates of SAE of interest for each of the six diseases are listed in table 2.

**Table 1:** Baseline characteristics of patients by specific diseases

	Rheumatoid arthritis	Psoriatic arthritis	Ankylosing spondylitis	Juvenile idiopathic arthritis	Psoriasis	Crohn’s disease	
N	12 345	837	1641	171	1819	2228	
Mean age, years	53.8	48.4	43.2	11.8	44.1	38.3	
Mean disease duration, years	10.6*	14.6*	11.1*	3.8	18.5	11.7*	
Female, %	79.1	47.4	27.7	78.9	32.3	61.3	
Median duration of exposure, years (range)	0.70 (0.04–9.03)	0.39 (0.04–3.53)	0.38 (0.04–3.04)	2.99 (0.04–4.50)	1.36 (0.04–4.01)	0.50 (0.04–4.42)	
On concomitant immunosuppressants, %	61.8	55.6	17.8	49.7	0.3	40.2	
On concomitant systemic steroids, %	58.6	19.1	15.4	21.6	1.2	35.3	
From US sites, %	21.4	25.3	8.8	51.5	53.3	57.3	

*Based on the following number of patients with available baseline disease duration information: rheumatoid arthritis, 11 984; psoriatic arthritis, 819; ankylosing spondylitis, 1640; Crohn’s disease, 1933.

**Table 2:** SAE of interest, events/100 patient-years, as of 15 April 2007

	Rheumatoid arthritis	Psoriatic arthritis	Ankylosing spondylitis	Juvenile idiopathic arthritis	Psoriasis	Crohn’s disease
N	12 345	837	1641	171	1819	2228
Exposure (patient-years)	18 284.3	997.5	1255.2	398.4	2424.7	2373.7
Serious infections	4.65	2.81	1.11	2.76	1.32	5.18
Tuberculosis	0.29	0.30	0	0	0.12	0.13
Opportunistic infections	0.09	0	0	0	0	0.08
Histoplasmosis	0.03	0	0	0	0	0
Malignancies excluding lymphoma and NMSC	0.76	0.30	0.08	0	0.49	0.46
Lymphoma	0.12	0.20	0.08	0	0	0.08
NMSC	0.17	0	0.08	0	0.12	0
Demyelinating disorder	0.05	0	0.08	0	0	0.13
Lupus-like syndrome	0.07	0	0	0	0	0.04
Congestive heart failure	0.23	0	0.16	0	0	0

NMSC, non-melanoma skin cancer; SAE, serious adverse event.

### Stability of safety profile in RA over time

Cumulative SAE rates from 2002, 2004, 2005 and 2006^31^ ^39^ ^40^ ^41^ were comparable to those from 2007: serious infections (4.6–5.1 vs 4.7/100 patient-years), tuberculosis (0.22–0.28 vs 0.29/100 patient-years), lymphomas (0.10–0.21 vs 0.12/100 patient-years), demyelinating disease (0.05–0.08 vs 0.05/100 patient-years) and lupus-like syndrome (0.05–0.10 vs 0.07/100 patient-years). Of these events, serious infections remained the most common.

### SAE of interest across six immune-mediated inflammatory diseases

#### Serious infections

**Figure 1:**
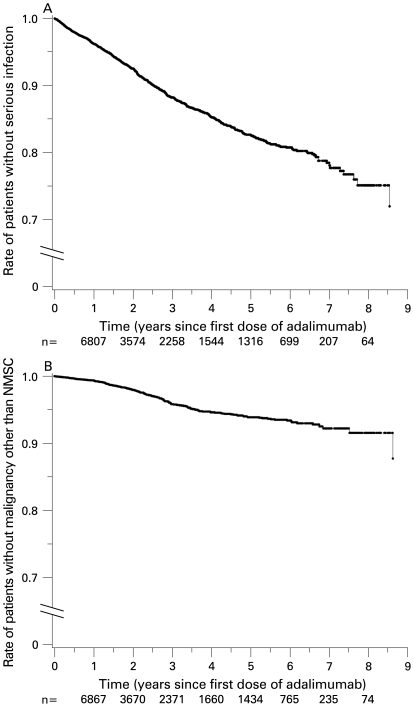
(A) Time to first serious infection, all adalimumab patients. (B) Time to first malignancy other than non-melanoma skin cancer (NMSC), all adalimumab patients. Overall scales are from 1.0 to 0.0; the portion of the scale from 0.7 to 1.0 has been emphasised for clarity. Numbers along the *x*-axis represent the numbers of patients who were at risk for each time point, ie, those still in the study who had not yet experienced the event.

The greatest rates of serious infections were observed in RA and CD clinical trials (table 2). The risk of patients experiencing their first serious infection was not greater at any one point during the course of therapy (fig 1). The most commonly reported serious infections were pneumonia for RA and abscess for CD (intra-abdominal or gastrointestinal tract related). Patients with early RA (disease duration <3 years, mean 0.7 years) had a serious infection rate of 2.76/100 patient-years compared with 4.91/100 patient-years in those with established RA.

Tuberculosis rates for each of the six diseases were similar, with the exception of AS and JIA, for which no cases of tuberculosis have been reported. Among patients with RA, extrapulmonary involvement was reported in 33 of the 53 tuberculosis cases.

#### Opportunistic infections

Most opportunistic infections reported in adalimumab clinical trials did not fulfil the regulatory criteria for SAE as determined by the investigators. Most commonly reported was oral candidiasis. In RA, 17 opportunistic infections (0.09/100 patient-years) considered to be SAE occurred: six cases of histoplasmosis; three cases of cytomegaloviral infections and one case each of coccidioidomycosis, toxoplasmosis, listeriosis, nocardiosis, aspergilloma, *Pneumocystis jiroveci* infection, oesophageal candidiasis and candida sepsis. In CD, two cases (0.08/100 patient-years) of opportunistic infections that were SAE occurred, one case each of nocardiosis and coccidioidomycosis. Opportunistic infections were infrequently reported among patients with other immune-mediated inflammatory diseases: six cases in PsA; eight in AS; one in JIA and five in psoriasis. Nearly all reported events were oral candidiasis; none were SAE (table 2). No cases of progressive multifocal leukoencephalopathy have been reported in adalimumab clinical trials.

#### Demyelinating disorders

Few cases of demyelinating disorders were reported during adalimumab clinical trials (table 2). Thirteen cases were reported in RA studies: six cases of multiple sclerosis; two Guillain–Barré syndrome; two optic neuritis; two non-specific demyelination and one optic nerve disorder. Ten (0.05/100 patient-years) were SAE. One optic neuritis event (0.08/100 patient-years), also an SAE, was reported during AS trials. Three cases of optic neuritis and one case of multiple sclerosis were reported in CD studies. Three (0.13/100 patient-years) were SAE. No demyelinating disorders were observed in JIA, PsA and psoriasis trials.

#### Lupus-like syndrome

Lupus-like syndrome was infrequent among adalimumab-treated patients (table 2). Thirty-five events in the lupus-like syndrome category occurred during RA trials, only 12 (0.07/100 patient-years) were SAE: six cases of lupus-like syndrome; three systemic lupus erythematosus; two cutaneous lupus erythematosus and one antiphospholipid antibody syndrome. Six events were reported in CD trials. Only one case (0.04/100 patient-years) of lupus-like syndrome was an SAE. One patient in the psoriasis clinical development programme developed cutaneous lupus erythematosus, which was not considered an SAE by the investigator. No cases were reported in PsA, AS and JIA trials. Most patients with RA or CD who reported lupus-like syndrome SAE presented with cutaneous manifestations or serositis. None had internal organ involvement (eg, nephritis).

### Adalimumab malignancy and mortality rates versus data from the general population

#### Malignancies

**Figure 2:**
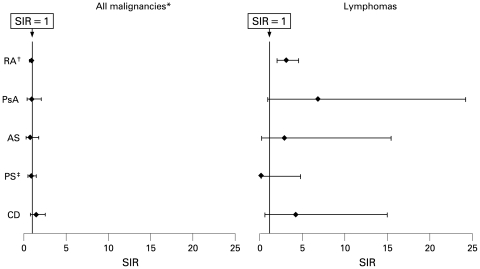
Standardised incidence rates (SIR) for all malignancies and lymphomas for rheumatoid arthritis (RA), psoriatic arthritis (PsA), ankylosing spondylitis (AS), psoriasis (Ps) and Crohn’s disease (CD). *All malignancies other than non-melanoma skin cancer. †Based on data from 12 344 patients. All other diseases included all the patients in the analysis. ‡No lymphomas were observed in psoriasis. No malignancies were observed in juvenile idiopathic arthritis.

The risk of malignancy was not greater at any one point during the course of adalimumab therapy (fig 1). The SIR by cancer site for patients enrolled in RA trials are included in supplemental table 1 (available online only). The SIR for malignancies in clinical trials for all diseases combined was 0.83 (95% CI 0.72 to 0.96). The SIR for lymphomas reported in fig 2 were based on 23 cases (19 cases of non-Hodgkin’s lymphoma and four cases of Hodgkin’s lymphoma) from RA, two from PsA, two from CD and one from AS trials (fig 2). No lymphomas were reported during JIA and psoriasis trials. The observed number of lymphoma cases was significantly greater than the expected number only in the RA trials (SIR 2.98; 95% CI 1.89 to 4.47). The rate for the early RA population was 0.09/100 patient-years compared with 0.12/100 patient-years in established RA.

**Figure 3:**
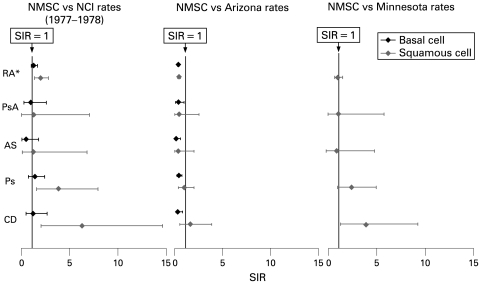
Standardised incidence rates (SIR) for non-melanoma skin cancer (NMSC) using three comparator databases. *Based on data from 12 344 patients. All other diseases included all the patients in the analysis. No malignancies were observed for juvenile idiopathic arthritis. AS, ankylosing spondylitis; CD, Crohn’s disease; NCI, National Cancer Institute; Ps, psoriasis; PsA, psoriatic arthritis; RA, rheumatoid arthritis.

The SIR for NMSC varied depending on which comparator database was employed (fig 3). Based on the NCI database, SIR (95% CI) for BCC (1.24 (1.01 to 1.51)) and SCC (1.97 (1.34 to 2.80)) for RA and SCC for CD (6.27 (2.02 to 14.6)) and psoriasis (3.84 (1.54 to 7.92)) were significantly greater than 1.0. These SIR were no longer significantly greater than 1.0 when either the Arizona or Minnesota rates were employed for comparison, with the exception of SCC for CD (3.97 (1.28 to 9.26)), based on the Minnesota database. The BCC and SCC SIR for all other diseases, regardless of the comparator database, did not demonstrate statistically significant differences between observed and expected numbers of cases (SIR <1 or 95% CI included 1.0). In clinical trials for all diseases considered collectively and in RA trials alone, other than lymphomas and NMSC, no other type of malignancy had a significantly greater incidence (SIR >1.0 and 95% CI did not include 1.0) compared with the general population (data not shown).

#### Mortality rates

**Figure 4:**
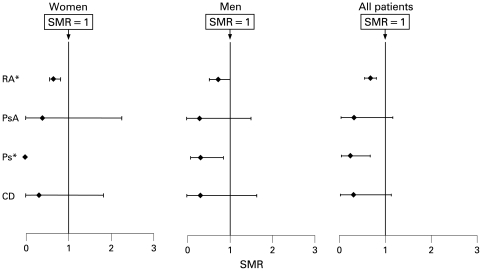
Standardised mortality rates (SMR) for rheumatoid arthritis (RA), psoriatic arthritis (PsA), psoriasis (Ps) and Crohn’s disease (CD). No deaths occurred in juvenile idiopathic arthritis or ankylosing spondylitis. *No deaths occurred among female patients with psoriasis.

SMR for patients treated with adalimumab for each of the six diseases, regardless of sex, were all less than 1.0 (ie, the number of deaths observed during treatment with adalimumab was less than what would be expected in the general population; fig 4). No deaths were reported in the JIA or AS clinical programmes.

## Discussion

This was the first comprehensive safety review of a TNF antagonist in all six of its approved disease indications. Our results extend the findings of previous long-term adalimumab safety reports for RA^31^ ^39^ ^40^ ^41^ by showing that the frequency of SAE of interest appeared stable after additional patient-years of exposure in RA, and by demonstrating that the safety profile of adalimumab in RA was comparable to PsA, AS, psoriasis, JIA and CD clinical trials.

Compared with the established and well-accepted overall safety profile of adalimumab in RA, data from clinical trials in other diseases yielded overall similar SAE rates. However, serious infections occurred more frequently in patients with RA or CD, whereas rates were lower in patients with the four other diseases evaluated. Potential reasons for these observations include inherent differences in risks of various AE between the diseases studied, differences in the severity and duration of disease, comorbidities and the use of concomitant medications (eg, corticosteroids or other immunosuppressants).^42^ ^43^ ^44^ ^45^ Serious infections occurred less frequently in patients with early RA than in the established RA population, suggesting that the risk of serious infections may be lower in patients who are treated earlier in their disease course.

The rate of serious infections in adalimumab RA trials was within the range reported in RA populations before the availability of TNF antagonists (3.1–9.6/100 patient-years)^42^ ^45^ and was comparable to that reported from long-term etanercept clinical trials (4.2/100 patient-years).^46^ Dixon *et al*^22^ did not find increased rates of serious infections in patients with RA in the anti-TNF cohort of the British Society for Rheumatology Biologics Registry when considering the entire duration of therapy, but noted an increased early risk (within the first 90 days of therapy commencement) compared with the disease-modifying antirheumatic drug cohort. Our analysis did not indicate that the occurrence of serious infections was clustered around any point in the course of adalimumab therapy, including the period immediately after the initiation of treatment.

As of this analysis, tuberculosis has not been reported for patients from adalimumab clinical trials in JIA or AS. There are two plausible explanations for this observation. First, only the AS development programme required re-screening for tuberculosis at year 2 and those who had seroconverted received prophylaxis. Second, on the basis of the positive effects of screening and prophylaxis, tuberculosis cases observed thus far with adalimumab therapy have generally been thought to have resulted from the reactivation of latent tuberculosis.^31^ Given that none of the patients in the JIA trial were purified protein derivative positive at study entry and that the JIA and AS populations are relatively young, tuberculosis reactivation was unlikely.

In 10 years of adalimumab RA clinical trials, SAE of interest other than serious infections continued to be of relatively low frequency and have not increased over time. SAE reports of demyelinating disorders, lupus-like syndrome and CHF have also not been observed in PsA, psoriasis and JIA. Other than the potential differences in predisposition or risks for these events between the diseases studied, another possible explanation is the smaller population sizes of these clinical development programmes compared with those for RA and CD.

Overall, the risk of malignancies with adalimumab in global clinical trials was not increased when compared with the general population. No malignancies were reported in JIA clinical trials. Risks associated with long-term use should be examined in future investigations that go beyond the scope of these long-term extension trials.

The observed number of cases of lymphoma in RA clinical trials was significantly greater than that expected for the general population. Patients with RA have an inherent twofold increased risk of developing lymphomas irrespective of therapy compared with the general population, especially those patients with greater disease activity for whom a 70-fold increased risk has been reported.^47^ ^48^ ^49^ Therefore, it is not unexpected to observe an SIR greater than 1.0 for patients with RA in the adalimumab clinical trial database compared with the general population.

Patients with RA have a disease-inherent increased risk of developing NMSC.^50^ Wolfe and Michaud^24^ reported an increased risk of NMSC (odds ratio 1.5; 95% CI 1.2 to 1.8) and melanoma (odds ratio 2.3; 95% CI 0.9 to 5.4) in patients with RA treated with biological agents (infliximab, etanercept, adalimumab, or anakinra) compared with patients not on biological therapy. For adalimumab, NMSC SIR varied according to the comparator database, with SIR generally greater using the NCI than the Arizona or Minnesota datasets. This is most likely because the Arizona and Minnesota datasets cover a later time period than the NCI, which demonstrated an increase in US skin cancer rates after the 1970s. Although the CI overlapped, the point estimate for the SCC SIR in psoriasis was consistently lower than for CD, regardless of the comparator database employed. Given the use of psoralen and ultraviolet A light (a known risk factor for NMSC)^51^ in patients with psoriasis and dermatologists’ heightened awareness of screening for these lesions, this suggests that adalimumab and phototherapy/topical therapies do not seem to act synergistically to increase the risk of NMSC in patients with psoriasis.

In terms of mortality rates, the SMR from adalimumab trials were less than 1.0 for all six diseases. No more deaths occurred with adalimumab than in the general population. The SMR in adalimumab-treated patients with RA was lower than the SMR previously reported for general RA populations naive to anti-TNF therapy in observational studies conducted in the USA, Canada and Spain.^52^ ^53^ ^54^ The SMR for PsA, CD and psoriasis from the adalimumab database were also lower than SMR previously published for these diseases.^55^ ^56^ ^57^

Several factors should be considered in drawing definitive conclusions about the SMR and SIR in adalimumab clinical trials. First, the inclusion/exclusion criteria of each study have probably selected a patient sample with fewer or less severe comorbidities. Furthermore, there is a possibility that those remaining in the study at the end of the follow-up period were healthier, confounding the seemingly stable results over time. Second, clinical trial participation also implies better follow-up and closer monitoring of patients. Third, the control of inflammation in RA with adalimumab may potentially lower some of the risks of mortality (ie, cardiovascular disease). Therefore, although these data provide information about the overall safety profile of adalimumab, conclusions beyond their primary intent in characterising safety should be made with caution.

Important insights about the safety of TNF antagonists may be gained from 10 years of adalimumab clinical trial experience across several immune-mediated inflammatory diseases in more than 19 000 patients. Although SAE such as tuberculosis, demyelinating disorders, malignancies and lupus-like syndromes require careful diligence, the most frequently reported events are still serious infections. Physicians and patients need to be vigilant for the signs and symptoms of infections when initiating or continuing biological therapy, including the implementation of appropriate tuberculosis screening before treatment. Our observations indicate that the safety profile of a drug such as adalimumab is probably influenced by a combination of drug-based toxicity, disease-inherent risks for certain SAE and baseline patient characteristics.

The adalimumab data presented in this report support its safety for long-term use and in patients across six different immune-mediated inflammatory diseases. Given the confirmed efficacy and substantial benefits of adalimumab in these conditions, the risk of therapy should be weighed against the risk of uncontrolled inflammatory disease and its long-term sequelae. The extent of the safety data generated by this analysis provides physicians with further information to enhance their benefit–risk discussions with patients.
